# Evaluation of a Multi-Component Phenotype Algorithm for Systemic Lupus Erythematosus across the PCORnet, OMOP, and i2b2 Common Data Models

**DOI:** 10.23889/ijpds.v9i5.2908

**Published:** 2024-09-18

**Authors:** Steven Tran, Luke Rasmussen, Jennifer Pacheco, Philip Silberman, Martin Borsje, Prasanth Nannapaneni, Daniel Schneider, Yuan Luo, Abel Kho, Rosalind Ramsey-Goldman, Theresa Walunas

**Affiliations:** 1 Northwestern University Feinberg School of Medicine

The aim of this study was to evaluate the performance of a computable phenotype for systemic lupus erythematosus (SLE) patients when it is ported from a local data warehouse to the i2b2, OMOP, and PCORnet CDMs.

We adapted the SLE phenotype to the Northwestern Medicine (NM) Enterprise Data Warehouse (EDW) and NU i2b2, OMOP, and PCORnet instances. Each of the phenotype’s 17 components were determined by a rules-based algorithm built on diagnosis, medication, lab, and procedure codes. We assessed the phenotype over 168 clinician-confirmed SLE patients and 100 healthy controls and calculated agreement between the EDW and CDMs for overall SLE classification, individual SLICC criteria membership, and individual code occurrence level aggregates - count, first and last date, lab values using Cohen’s kappa (Cκ) and intraclass correlation coefficients (ICC).

For overall SLE classification, agreement of the OMOP and PCORnet datamarts with the EDW was high (Cκ 0.928, 0.802, respectively) while that of i2b2 was low (Cκ 0.328). For the panel of SLICC criteria, OMOP had high agreement for 14/17 SLICC criteria (Cκ 0.792-1.000). PCORnet had high agreement for 13/17 criteria (Cκ 0.708-1.000). i2b2 had high agreement for 10/17 criteria (Cκ 0.813-1.000) with the exception of lab-based criteria.

While agreement between the EDW and CDMs at the level of individual codes was relatively poor, agreement at the levels of overall SLE classification and individual SLICC criteria were reasonably high for OMOP and PCORnet, suggesting that inconsistencies at a micro, code level become less apparent at the macro, phenotype level.

